# Incidence and treatment of complex regional pain syndrome after surgery: analysis of claims data from Germany

**DOI:** 10.1097/PR9.0000000000001210

**Published:** 2024-11-20

**Authors:** Karolin Teichmüller, Norman Rose, Johannes Dreiling, Daniel Schwarzkopf, Winfried Meißner, Heike L. Rittner, Gudrun Kindl

**Affiliations:** a Department of Anaesthesiology, Intensive Care, Emergency and Pain Medicine, Centre for Interdisciplinary Pain Medicine, University Hospital Würzburg, Würzburg, Germany; b Department of Psychology, University of Würzburg, Würzburg, Germany; c Department of Anaesthesiology and Intensive Care Medicine, Section Pain Therapy, Friedrich-Schiller-University Jena, University Hospital Jena, Jena, Germany

**Keywords:** Complex regional pain syndrome, Incidence, Pain therapy, Claims data, Health services research

## Abstract

**Introduction:**

Complex regional pain syndrome (CRPS) is a rare complication after limb injuries. Early recognition of the symptomatology and interdisciplinary interventions are essential to prevent long-term disability and pain.

**Objective:**

This article presents results on the incidence of CRPS after surgery in Germany and treatments used by patients with CRPS, using claims data from the BARMER, a German nationwide health care insurance.

**Methods:**

A total of N = 85,862 BARMER patients with inpatient surgery on the upper or lower limb in 2018 were included. Patients with CRPS were identified by documented *International Statistical Classification of Diseases and Related Health Problems-10* diagnosis within 12 months after surgery. For the same period, medication and nonpharmaceutical therapies for inpatient and outpatient care were assessed.

**Results:**

The overall incidence of CRPS within 12 months after surgery was 0.34%. With 0.60%, the incidence of CRPS after surgeries of the upper limb was 3 times higher than after lower-limb surgeries (0.20%). Women were more frequently affected, and most patients were between 50 and 70 years old. About 80% to 90% of patients with CRPS received physiotherapy and nonopioid pain medication within 12 months after surgery. Approximately 40% to 50% were treated with opioids and/or antineuropathic medication. Cortisone, bisphosphonates, pain therapy, and occupational therapy were rarely claimed.

**Conclusion:**

We found a low incidence of CRPS after various types of surgeries. Although previous research has focused on distal radius fractures and ankle surgery, our data suggest that clinicians should be aware of CRPS after other types of surgeries as well. Real-world treatment of CRPS does not reflect recommendations in clinical practice guidelines.

## Abstract 2

Supplemental Digital Content is Available in the Text.

Complex regional pain syndrome occurs rarely (<1%) but is not limited to specific surgical procedures. Patients in Germany are rarely supplied with pain therapy and occupational therapy.

## 1. Introduction

Complex regional pain syndrome (CRPS) is a chronic pain disorder that occurs as a rare complication after fractures or soft-tissue injuries of the upper or lower extremity. The clinical diagnostic criteria include persisting pain that is disproportionate to any inciting event, sensory alterations (hyperesthesia and allodynia), vasomotor symptoms, motor dysfunction, and trophic changes.^15^

Population-based studies in the United States and the Netherlands report incidence rates of 5.5 and 26.2 per 100,000 person-years, respectively.^8,24^ Women are affected 3 to 4 times more frequently than men, and CRPS occurs 1.5 to 2 times more often at the upper compared with the lower limb. Most studies indicate an incidence peak in patients around 50 years of age.^8,21,24^

Fractures represent the most common trigger, which is why most studies report CRPS incidence rates after different types of fractures, eg, 0.36% to 1% after distal radius fractures,^11,14,21^ 0.3% after foot or ankle fractures,^5^ and 4 to 14% after a wrist fracture.^23^ Rolls et al.^23^ observed lower rates in conservatively managed wrist fractures, which suggests that surgical procedures might represent an additional risk factor for developing CRPS. Furthermore, CRPS incidence seems to be higher in patients with motor or sensory nerve injury.^10^ However, the pathogenesis of CRPS remains poorly understood.^13^

Although rare, CRPS causes significant individual burden, including prolonged pain, loss of motor function, and compromises to work status.^7,22^ For a favourable prognosis, early recognition of the symptoms and intervention is believed to be crucial.^3^ Treatment guidelines recommend multidisciplinary care that includes pharmaceutical and nonpharmaceutical treatments, especially oral medication and physiotherapy and occupational therapy.^4^ However, high-certainty evidence for the effectiveness of most pharmaceutical interventions for CRPS is still lacking.^12^ Psychological comorbidities should be evaluated and, if present, targeted by psychotherapy. Nevertheless, short-term access to professional diagnostics and therapy is not always straightforward. Thus, the aim of this evaluation is to describe the incidence of CRPS after different types of surgeries and the health care situation of patients with CRPS in Germany using claims data. We expected to find incidences comparable with those in other Western countries,^8,21^ with women and middle-aged patients being more frequently affected, and distal radius fractures bearing the highest risk of developing CRPS compared with other limb surgeries. Furthermore, it was hypothesised that real-world health care for patients with CRPS should mirror guideline-based treatment recommendations.

## 2. Methods

This study was conducted as part of the LOPSTER project,^25^ a study on the long-term outcome of perioperative pain therapy funded by the German Innovations Fund of the Federal Joint Committee in Germany (G-BA, FKz 01VSF19019). LOPSTER was approved by the ethics committee of the University Hospital Jena (approval number 2020-1952-Daten). The analyses are based on anonymised data from the BARMER, a large German statutory health insurance company with approximately 8.7 million members.^1^

In Germany, health insurance is mandatory for all residents. This can be through statutory or private health insurance. Most Germans are covered by statutory health insurance, which is funded through contributions from employees and employers. Individuals can choose from various providers, including the BARMER. For the present analyses, BARMER patients were included if they had undergone inpatient surgery of the upper or lower extremity in 2018. This year was chosen to ensure that treatments during a 12-month follow-up period were unaffected by the SARS-CoV-2 epidemic, which reached Germany in January 2020. If patients had more than one hospital stay with a surgical procedure, only the first stay was chosen and defined as the index stay. Surgical procedures were classified into 20 groups, comprising 9 for upper-limb surgery and 11 for lower-limb surgery. To control for a pre-existing diagnosis of CRPS, patients were identified, for whom an *International Statistical Classification of Diseases and Related Health Problems-10* code for CRPS had been recorded in the 12 months before surgery. The codes and conditions used to identify CRPS are listed in Table 1.

**Table 1:** *ICD-10-GM* (German modification) codes and conditions for identification of complex regional pain syndrome.

G90.5x: CRPS type I
G90.50: CRPS type I of upper limb; G90.51: CRPS type I of lower limb; G90.59: CRPS type I, site not specified
G90.6x: CRPS type II
G90.60: CRPS type II of upper limb; G90.61: CRPS type II of lower limb; G90.69: CRPS type II, site not specified
G90.7x: CRPS type not specified
G90.70: CRPS of upper limb, type not specified; G90.71: CRPS of lower limb, type not specified; G90.79: CRPS, type and site not specified
M89.0x: algoneurodystrophy*
M89.00: algoneurodystrophy, multiple sites; M89.01: algoneurodystrophy, shoulder; M89.02 algoneurodystrophy, upper arm; M89.03: algoneurodystrophy, forearm; M89.04: algoneurodystrophy: hand; M89.05: algoneurodystrophy, pelvis and thigh; M89.06: algoneurodystrophy, lower leg; M89.07 algoneurodystrophy, ankle and foot; M89.08: algoneurodystrophy, other site; M89.09: algoneurodystrophy: site not specified

* This is an outdated term that should not be used to diagnose CRPS. However, it is still rarely used in general practice, and therefore, it was included.

CRPS, complex regional pain syndrome.

For a period of 12 months after surgery, physiotherapy and occupational therapy were recorded using settlement data from the specific health care professionals; pain therapy and psychotherapy were identified through the respective accounting codes for reimbursement, whereas medication was recorded by prescriptions using the WHO Anatomical Therapeutic Chemical classification. Definitions of variables can be found in the supplemental content, http://links.lww.com/PR9/A262. The incidence of CRPS was calculated stratified by type of surgery, sex, and age groups by decades. The proportions of patients with CRPS receiving specific treatments or medications were calculated, as well as group comparisons (χ^2^ tests) and odds ratios (OR) regarding health care for patients with vs without CRPS. It is important to note, however, that claims data do not allow for a specific assignment of a certain treatment to the CRPS diagnosis. All analyses were performed using *R*, and figures were created using Excel.

## 3. Results

**Figure 1.**
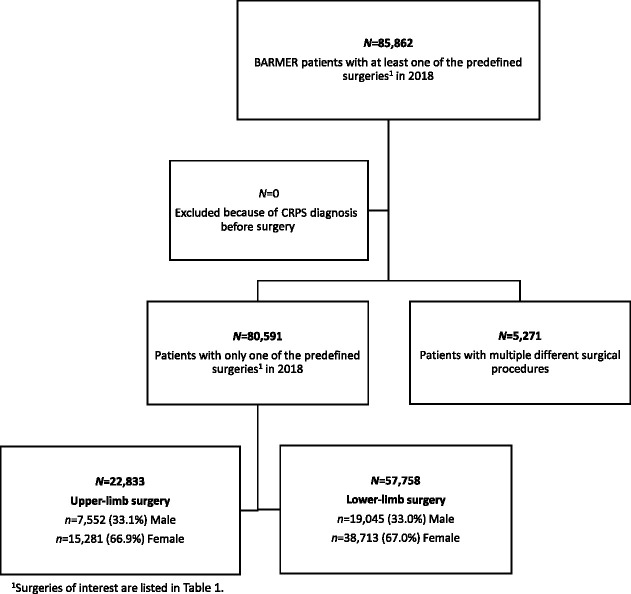
Flow chart with sample size and subgroups.

As illustrated in Figure 1, more than 85,000 BARMER patients underwent at least one of the previously defined surgeries in 2018. None of these patients had been diagnosed with CRPS in the 12 months before the procedure. A subgroup of 5,271 patients had received other surgical procedures in addition to those defined for inclusion.

Within 12 months after surgery, N = 288 patients (0.34%) of the initial sample received a first-time diagnosis of CRPS. Of those with only 1 surgery, n = 251 patients (0.31%) developed CRPS during the following year. The incidence of CRPS after upper-limb surgery (0.60%, n = 138) was 3 times higher than after lower-limb surgery (0.20%, n = 113). After having had multiple surgical procedures, n = 37 patients (0.70%) were diagnosed with CRPS.

We also analysed the effects of age, sex, and type of surgery as potential risk factors for CRPS after surgery. These calculations are based on the subgroup with one of the defined surgeries only to allow for a unique classification.

### 3.1. Demography of the cohort in line with known distributions

The association between CRPS and age differed slightly between upper- and lower-limb surgery. Complex regional pain syndrome after upper-limb surgery was most incident in patients aged 50 to 70 years (*M* = 62.5 years, *SD* ± 11.4), whereas the peak for CRPS after lower-limb surgery was between 40 to 50 years (*M* = 59.4 years, *SD* ± 13.5). A detailed presentation of the percentage of CRPS cases in relation to the total number of patients per age decade can be found in the supplemental content, http://links.lww.com/PR9/A262.

Among females undergoing upper-limb surgery, 0.78% developed CRPS compared with 0.25% among males. For lower-limb surgery, the difference was smaller, with an incidence of CRPS of 0.24% among females and 0.11% among males.

### 3.2. Type of surgery

The incidence rates of CRPS by surgical groups are shown in Table 2.

**Table 2:** Incidence of complex regional pain syndrome after different types of surgery.

Type of surgery	N	CRPS incidence
Upper-limb surgery		
Open reposition distal radius	6,581	77 (1.17%)
Hand resection arthroplasty	566	6 (1.06%)
Hand tendon, ligament, and fascia repair	2,573	21 (0.82%)
Partial shoulder joint replacement proximal humerus	129	1 (0.78%)
Decompression or neurolysis of hand or arm nerves	930	7 (0.75%)
Shoulder joint replacement, both conventional and inverse	1,421	4 (0.28%)
Shoulder operation	8,456	18 (0.21%)
Open reposition proximal humerus	2,057	4 (0.19%)
Arthrodesis interphalangeal	120	0 (0.00%)
Total for upper-limb surgery	22,833	138 (0.60%)
Lower-limb surgery		
Arthrodesis ankle joint open	412	4 (0.97%)
Arthroscopy ankle joint operation	513	4 (0.78%)
Open reposition distal fibula and tibia	4,543	27 (0.59%)
Foot surgery	3,405	17 (0.50%)
Knee joint replacement revision	984	4 (0.41%)
Arthroscopy knee operation	11,189	18 (0.16%)
Hip joint replacement revision	1,264	2 (0.16%)
Hip joint replacement	19,164	25 (0.13%)
Toe amputation	1,525	2 (0.13%)
Removal material femur	913	1 (0.11%)
Knee joint replacement	13,846	9 (0.07%)
Total for lower-limb surgery	57,758	113 (0.20%)

CRPS, complex regional pain syndrome.

The highest incidence of about 1% occurred after surgery for distal radius fracture, hand arthroplasty, or arthrodesis of the ankle. All other types of surgeries, with the exception of arthrodesis of interphalangeal joints, were also associated with a risk, albeit small, of developing CRPS.

### 3.3. Treatment of complex regional pain syndrome after limb surgery

**Figure 2.**
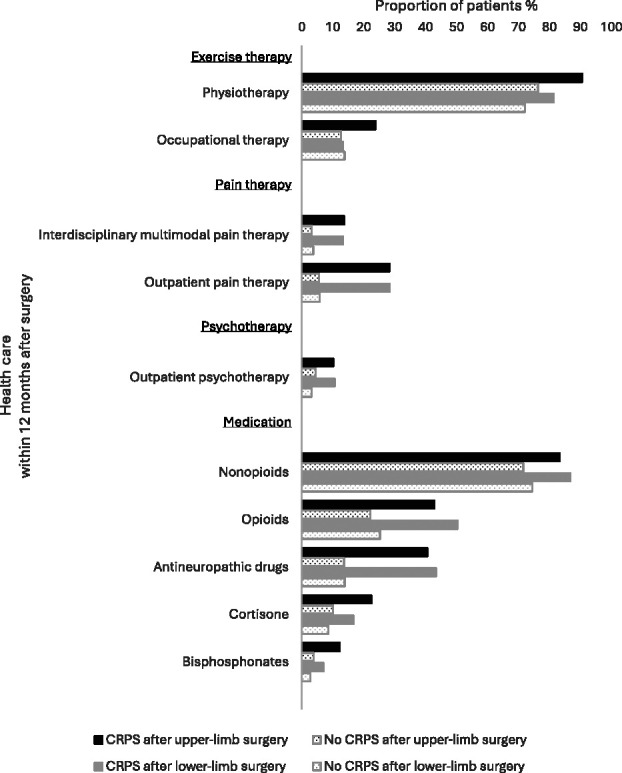
Health care for patients with and without CRPS within 1 year of limb surgery. Overall, physiotherapy and nonopioid medication were frequently claimed after limb surgery. For all treatments, the proportion of patients with CRPS and controls differed significantly, except for occupational therapy after lower-limb surgery. CRPS, complex regional pain syndrome.

Most patients with CRPS received physiotherapy and nonopioid pain medication within 12 months after surgery (Fig. 2). Almost half were treated with opioids and/or antineuropathic drugs. Only about 20% of patients with CRPS were prescribed cortisone, and even fewer were prescribed bisphosphonates. Occupational therapy, interdisciplinary pain therapy, and outpatient pain therapy were claimed by less than 30%.

During the 12-month follow-up period, a significantly higher proportion of patients with CRPS after upper-limb surgery received the investigated treatments compared with patients without CRPS after upper-limb surgery (all χ^2^(1) ≥ 8.75, *P* < 0.01). Also after lower-limb surgery, all treatments were claimed more frequently by patients with CRPS than controls (all χ^2^(1) ≥ 4.45, *P* < 0.05), except for occupational therapy (χ^2^(1) *=* 0.00, *ns*).

As shown in Table 3, the odds of receiving pain therapy and antineuropathic drugs were 4 to 6.5 times higher in patients with CRPS than in controls, suggesting that these treatments can most likely be attributed to the CRPS condition. The odds ratios (ORs) for bisphosphonates and cortisone were comparable with those for opioids. The lowest ORs occurred for exercise therapy after lower-limb surgery, indicating a rather unspecific usage of physiotherapy and occupational therapy in this subgroup.

**Table 3:** Odds ratios for receiving treatment within 12 months after surgery.

Treatment	Upper-limb surgery			Lower-limb surgery		
	*N*_CRPS_	*N*_noCRPS_	OR [95% CI]	*N*_CRPS_	*N*_noCRPS_	OR [95% CI]
Exercise therapy						
Physiotherapy	125 (90.6%)	17,324 (76.3%)	2.98 [1.75, 5.55]	92 (81.4%)	41,541 (72.1%)	1.70 [1.08, 2.80]
Occupational therapy	33 (23.9%)	2,875 (12.7%)	2.17 [1.44, 3.17]	15 (13.3%)	7,972 (13.8%)	0.95 [0.53, 1.59]
Pain therapy						
Interdisciplinary multimodal pain therapy	19 (13.8%)	740 (3.3%)	4.74 [2.82, 7.54]	15 (13.3%)	2,154 (3.7%)	3.94 [2.20, 6.59]
Outpatient pain therapy	39 (28.3%)	1,270 (5.6%)	6.65 [4.52, 9.58]	32 (28.3%)	3,318 (5.8%)	6.47 [4.23, 9.65]
Psychotherapy						
Outpatient psychotherapy	14 (10.1%)	1,025 (4.5%)	2.39 [1.31, 4.02]	12 (10.6%)	1876 (3.3%)	3.53 [1.84, 6.17]
Medication						
Nonopioids	115 (83.3%)	16,245 (71.6%)	1.99 [1.29, 3.19]	98 (86.7%)	42,923 (74.5%)	2.24 [1.34, 4.02]
Opioids	59 (42.8%)	5,026 (22.2%)	2.63 [1.86, 3.68]	57 (50.4%)	14,565 (25.3%)	3.01 [2.08, 4.36]
Antineuropathic drugs	56 (40.6%)	3,101 (13.4%)	4.32 [3.05, 6.06]	49 (43.4%)	8,006 (13.9%)	4.75 [3.26, 6.88]
Cortisone	31 (22.5%)	2,275 (10.0%)	2.60 [1.71, 3.84]	19 (16.8%)	4,942 (8.6%)	2.16 [1.28, 3.45]
Bisphosphonates	17 (12.3%)	853 (3.8%)	3.60 [2.08, 5.83]	8 (7.1%)	1,575 (2.3%)	2.71 [1.21, 5.23]

CRPS, complex regional pain syndrome; OR, odds ratios.

## 4. Discussion

Using claims data, we found a low rate of CRPS after limb surgery when groups of surgical procedures were chosen as the first criterion. In addition to previous literature, we were able to show that not only distal radius fractures or wrist and ankle surgery are at risk of developing CRPS but also other groups of shoulder or knee surgery. Upper-limb surgery and multiple and revision surgeries were associated with higher CRPS incidence rates. This should be considered in clinical aftercare and when planning future studies for the early detection of CRPS. Also, the anatomical or physiological conditions that might contribute to the lower incidence of CRPS after knee surgery compared with ankle surgery should be investigated in the future.

Reasons for the low incidence in our data could be missed or delayed diagnosis in real-world conditions. General practitioners, who are the first points of contact for patients after hospital discharge, are probably not very familiar with the rare condition of CRPS. It may be that, in general practice, especially less severe cases with a spontaneous favorable course are less well identified than in prospective studies. Also, CRPS without previous surgery is not included in our data.

Fortunately, we found that more than 80% of patients with CRPS had access to physiotherapy, which is considered essential for functional restauration.^17^ Occupational therapy and interdisciplinary pain therapy were less frequently claimed. Possible reasons for this could be undertreatment, less necessity, or difficulties for patients to access these services within a 12-month period. Antineuropathic pain medication such as gabapentinoids or antidepressants, especially amitriptyline, are used frequently in CRPS^26^ and were prescribed to about 40% of patients with CRPS. Although recommended in the German CRPS guideline,^4^ bisphosphonates and cortisone were rarely used. Other nonsteroidal anti-inflammatory drugs, here nonopioid pain medication, were applied far more often. Opioids were prescribed just as frequently as antineuropathic medications, although they are recommended as second- or third-line therapy in low doses.^16^ However, evidence-based pharmaceutical recommendations are lacking,^12^ and the evaluation of treatment efficacy is beyond the scope of this article.

Most patients claimed physiotherapy and nonopioid medication, reflecting a broad indication of these treatments after limb surgery. Pain therapy and antineuropathic drugs seem to be used specifically for CRPS, whereas occupational therapy, cortisone, and bisphosphonates could be applied more specifically. According to our findings, especially treatment of lower-limb CRPS does not follow guideline recommendations.

However, these conclusions must be drawn tentatively. It remains unclear whether the prescription of a particular drug, as well as physiotherapy or occupational therapy, can be attributed to the diagnosis of CRPS or some other medical condition that we did not assess. The same applies to psychotherapy. In our sample, 10% of the patients with CRPS received outpatient psychotherapy within 1 year of surgery. Psychotherapy is indicated for patients with CRPS with high levels of anxiety, pain-related fear, and disability.^2^ However, given a 12-month prevalence of approximately 30% for mental disorders in the general German population,^18–20^ patients in our cohort may have received psychotherapy because of other, unrelated mental issues. An additional limitation is that we could not include CRPS without previous surgery. Complex regional pain syndrome can occur spontaneously or after minor injuries that do not require surgical intervention.^6,9^ To estimate the incidence of these cases as well, an additional control group without limb surgery during the study period would have been necessary. As it is not feasible to generate a healthy control group from claims data, prospective studies are needed to overcome this limitation. Furthermore, the inclusion of algoneurodystrophy in our analysis needs to be discussed. This was performed to increase sensitivity, although algoneurodystrophy is an outdated term that should not be used to describe CRPS. As a general limitation to the secondary analysis of claims data, we must acknowledge that the coding of diagnoses and procedures for billing purposes depends on coding practices, patients' help-seeking behavior, as well as the specific composition of health insurance policyholders.

Analyzing claims data from a large German health insurance company, we found a low incidence of CRPS (<1%) after various types of surgeries. Although previous research has focused mainly on distal radius fractures^11,21^ and ankle surgery,^5^ our data suggest that CRPS is not limited to a specific type of injury or surgery. Particularly after multiple or revision surgeries, clinicians from different medical disciplines should be aware of CRPS when providing follow-up care. Low-threshold access to adequate treatment, especially occupational therapy, and pain therapy, should be provided promptly to target pain and disability, thereby increasing the chances of a favourable prognosis.^7^

In summary, we found a very low incidence in real-world data compared with prospective studies. Correspondingly, the treatment reality demonstrates that more information to all stakeholders about guideline-based diagnostics and treatment of CRPS is necessary. Future studies can use these data as a basis for prevention and early detection of CRPS.

## Disclosures

H.R. received consultant fees from Gruenenthal and Orion and financial support for a study by Algiax. W.M. received consultant fees from Tafalgie, Kyowa, Mundipharma, Grünenthal, Ethypharm, and Spectrum Therapeutics. His institution received research support from European Commission, Gemeinsamer Bundesausschuss (GBA), Medtronic, Pfizer, Mundipharma, Grünenthal, and Vertanical. K.T., N.R., J.D., D.S., and G.K. have no conflicts of interest.

## Supplemental digital content

Supplemental digital content associated with this article can be found online at http://links.lww.com/PR9/A262.
