# Supplementary material for: Incidence and treatment of complex regional pain syndrome after surgery: analysis of claims data from Germany
Source: Pain Rep. 2024 Nov 20;9(6):e1210. doi: 10.1097/PR9.0000000000001210 (PMC11581752; doi:10.1097/PR9.0000000000001210)

Supplement 1. Operationalization of study variables.

UPPER LIMB SURGERY (ULS)

Partial shoulder joint replacement proximal humerus

OPS[1] code 5-824.0

Shoulder joint replacement, both conventional and invers

OPS[1] code 5-824.2

Shoulder operation

OPS[1] codes 5-814, 5-810.00, 5-810.01, 5-810.10, 5-810.11, 5-810.20, 5-810.21, 5-810.30, 5-810.31, 5-810.40, 5-810.41, 5-810.50, 5-810.51, 5-810.60, 5-810.61, 5-810.70, 5-810.71, 5-810.80, 5-810.81, 5-810.90, 5-810.91, 5-810.x0, 5-810.x1, 5-811.00, 5-811.01, 5-811.10, 5-811.11, 5-811.20, 5-811.21, 5-811.30, 5-811.31, 5-811.40, 5-811.41, 5-811.x0, 5-811.x1, 5-812.00, 5-812.01, 5-812.30, 5-812.31, 5-812.80, 5-812.81, 5-812.90, 5-812.91, 5-812.a0, 5-812.a1, 5-812.e0, 5-812.e1, 5-812.f0, 5-812.f1, 5-812.g0, 5-812.g1, 5-812.h0, 5-812.h1, 5-812.k0, 5-812.k1, 5-812.m0, 5-812.m1, 5-812.x0, 5-812.x1, 5-819.00, 5-819.01, 5-819.10, 5-819.11, 5-819.20, 5-819.21, 5-819.x0, 5-819.x1, 5-805

Open reposition proximal humerus

OPS[1] codes 5-793.01, 5-793.11, 5-793.21, 5-793.31, 5-793.41, 5-793.51, 5-793.61, 5-793.71, 5-793.81, 5-793.91, 5-793.a1, 5-793.b1, 5-793.c1, 5-793.e1, 5-793.g1, 5-793.h1, 5-793.k1, 5-793.m1, 5-793.n1, 5-793.x1, 5-794.01, 5-794.11, 5-794.21, 5-794.31, 5-794.41, 5-794.51, 5-794.61, 5-794.71, 5-794.81, 5-794.a1, 5-794.b1, 5-794.c1, 5-794.e1, 5-794.g1, 5-794.h1, 5-794.k1, 5-794.m1, 5-794.n1, 5-794.x1

Open reposition distal radius

OPS[1] codes 5-793.06, 5-793.16, 5-793.26, 5-793.36, 5-793.46, 5-793.56, 5-793.66, 5-793.76, 5-793.86, 5-793.96, 5-793.a6, 5-793.b6, 5-793.c6, 5-793.e6, 5-793.g6, 5-793.h6, 5-793.k6, 5-793.m6, 5-793.n6, 5-793.x6, 5-794.06, 5-794.16, 5-794.26, 5-794.36, 5-794.46, 5-794.56, 5-794.66, 5-794.76, 5-794.86, 5-794.a6, 5-794.b6, 5-794.c6, 5-794.e6, 5-794.g6, 5-794.h6, 5-794.k6, 5-794.m6, 5-794.n6, 5-794.x6

Hand resection arthroplasty

OPS[1] code 5-847

Hand tendon, ligament and fascia repair

OPS[1] codes 5-840, 5-841, 5-842

Arthrodesis interphalangeal

OPS[1] codes 5-846.4, 5-846.5, 5-846.6, 5-846.7

Decompression or neurolysis hand or arm nerves

OPS[1] codes 5-056.3, 5-056.4

LOWER LIMB SURGERY (LLS)

Toe amputation

OPS[1] codes 5-865.7, 5-865.8, 5-865.9

Hip joint replacement

OPS[1] code 5-820

Hip joint replacement revision

OPS[1] code 5-821

Removal material femur

OPS[1] code 5-787.0e, 5-787.0f, 5-787.0g, 5-787.0h, 5-787.1e, 5-787.1f, 5-787.1g, 5-787.1h, 5-787.2e, 5-787.2f, 5-787.2g, 5-787.2h, 5-787.3e, 5-787.3f, 5-787.3g, 5-787.3h, 5-787.4e, 5-787.4f, 5-787.4g, 5-787.4h, 5-787.5e, 5-787.5f, 5-787.5g, 5-787.5h, 5-787.6e, 5-787.6f, 5-787.6g, 5-787.6h, 5-787.7e, 5-787.7f, 5-787.7g, 5-787.7h, 5-787.8e, 5-787.8f, 5-787.8g, 5-787.8h, 5-787.9e, 5-787.9f, 5-787.9g, 5-787.9h, 5-787.ce, 5-787.cf, 5-787.cg, 5-787.ch, 5-787.ee, 5-787.ef, 5-787.eg, 5-787.eh, 5-787.ge, 5-787.gf, 5-787.gg, 5-787.gh, 5-787.ke, 5-787.kf, 5-787.kg, 5-787.kh, 5-787.me, 5-787.mf, 5-787.mg, 5-787.mh, 5-787.ne, 5-787.nf, 5-787.ng, 5-787.nh, 5-787.pe, 5-787.pf, 5-787.pg, 5-787.ph, 5-787.xe, 5-787.xf, 5-787.xg, 5-787.xh

Knee joint replacement

OPS[1] code 5-822

Knee joint replacement revision
OPS[1] code 5-823
Open reposition distal fibula and tibia
OPS[1] codes 5-793.0n, 5-793.1n, 5-793.2n, 5-793.3n, 5-793.4n, 5-793.5n, 5-793.6n, 5-793.7n, 5-793.8n, 5-793.9n, 5-793.an, 5-793.bn, 5-793.cn, 5-793.en, 5-793.gn, 5-793.hn, 5-793.kn, 5-793.mn, 5-793.nn, 5-793.xn, 5-794.0n, 5-794.1n, 5-794.2n, 5-794.3n, 5-794.4n, 5-794.5n, 5-794.6n, 5-794.7n, 5-794.8n, 5-794.an, 5-794.bn, 5-794.cn, 5-794.en, 5-794.gn, 5-794.hn, 5-794.kn, 5-794.mn, 5-794.nn, 5-794.xn, 5-793.0r, 5-793.1r, 5-793.2r, 5-793.3r, 5-793.4r, 5-793.5r, 5-793.6r, 5-793.7r, 5-793.8r, 5-793.9r, 5-793.ar, 5-793.br, 5-793.cr, 5-793.er, 5-793.gr, 5-793.hr, 5-793.kr, 5-793.mr, 5-793.nr, 5-793.xr, 5-794.0r, 5-794.1r, 5-794.2r, 5-794.3r, 5-794.4r, 5-794.5r, 5-794.6r, 5-794.7r, 5-794.8r, 5-794.ar, 5-794.br, 5-794.cr, 5-794.er, 5-794.gr, 5-794.hr, 5-794.kr, 5-794.mr, 5-794.nr, 5-794.xr
Arthrodesis ankle joint open
OPS[1] codes 5-808.7, 5-808.8
Foot surgery
OPS[1] codes 5-808.b, 5-788.00
Arthroscopy knee operation
OPS[1] code 5-810.0h, 5-810.1h, 5-810.2h, 5-810.3h, 5-810.4h, 5-810.5h, 5-810.6h, 5-810.7h, 5-810.8h, 5-810.9h, 5-810.xh, 5-811.0h, 5-811.1h, 5-811.2h, 5-811.3h, 5-811.4h, 5-811.xh, 5-812.0h, 5-812.3h, 5-812.8h, 5-812.9h, 5-812.ah, 5-812.eh, 5-812.fh, 5-812.gh, 5-812.hh, 5-812.kh, 5-812.mh, 5-812.xh, 5-819.0h, 5-819.1h, 5-819.2h, 5-819.xh, 5-812.5, 5-813
Arthroscopy ankle joint operation
OPS[1] codes 5-810.0k, 5-810.0m, 5-810.1k, 5-810.1m, 5-810.2k, 5-810.2m, 5-810.3k, 5-810.3m, 5-810.4k, 5-810.4m, 5-810.5k, 5-810.5m, 5-810.6k, 5-810.6m, 5-810.7k, 5-810.7m, 5-810.8k, 5-810.8m, 5-810.9k, 5-810.9m, 5-810.xk, 5-810.xm, 5-811.0k, 5-811.0m, 5-811.1k, 5-811.1m, 5-811.2k, 5-811.2m, 5-811.3k, 5-811.3m, 5-811.4k, 5-811.4m, 5-811.xk, 5-811.xm, 5-812.0k, 5-812.0m, 5-812.3k, 5-812.3m, 5-812.8k, 5-812.8m, 5-812.9k, 5-812.9m, 5-812.ak, 5-812.am, 5-812.ek, 5-812.em, 5-812.fk, 5-812.fm, 5-812.gk, 5-812.gm, 5-812.hk, 5-812.hm, 5-812.kk, 5-812.km, 5-812.mk, 5-812.mm, 5-812.xk, 5-812.xm, 5-819.0k, 5-819.0m, 5-819.1k, 5-819.1m, 5-819.2k, 5-819.2m, 5-819.xk, 5-819.xm

THERAPY

Physical therapy
Services invoiced by occupational group 225 and/or 240 (physiotherapists)
Occupational therapy
Services invoiced by occupational group 227 (occupational therapists)
Interdisciplinary multimodal pain therapy
OPS[1] codes 8-91, 8-91c
Outpatient pain therapy
EBM[2] codes 30700, 30701, 30702, 30704, 30705, 30706, 30708, 30710, 30712, 30724, 30731, 30760
Outpatient psychotherapy
EBM[2] codes 35150, 35151, 35152, 35401, 35402, 35405, 35411, 35412, 35415, 35421, 35422, 35425, 35431, 35432, 35435

MEDICATION

Non opioids
ATC codes N02BA01-16, N02BA19, N02BA20, N02BA51, N02BA55, N02BA57, N02BA59, N02BA65, N02BA71, N02BA75, N02BA77, N02BA79, N02BB01-06, N02BB51-56, N02BB71-74, N02BB76, N02BE01, N02BE03-05, N02BE51, N02BE53, N02BE54, N02BE61, N02BE71, N02BE73, N02BE74, N02BG02-12, M01AA01-03, M01AA05-07, M01AA51-53, M01AB01-17, M01AB19, M01AB51, M01AB55, M01AB68, M01AB69, M01AC01-06, M01AC56, M01AE01-18, M01AE20, M01AE51-53, M01AE56, M01AG01-04, M01AG06, M01AH01-07, M01AX01, M01AX02, M01AX04, M01AX05, M01AX07, M01AX12-14, M01AX17, M01AX18, M01AX21-27, M01AX55, M01AX68, M01BA01-08

Opioids

ATC codes N02AJ13-15, N02AX01, N02AX02, N02AX05, N02AX51, N02AA01, N02AA02-05, N02AA10, N02AA11, N02AA51, N02AA53, N02AA55-57, N02AB01-03, N02AB07, N02AB52, N02AB72, N02AC01, N02AC03-05, N02AC54, N02AC74, N02AD01, N02AD02, N02AE01, N02AF01, N02AF02, N02AG01-04, N02AG04, N02AJ17-19, N02AX03, N02AX06, N02AA08, N02AA58, N02AA59, N02AA79, N02AJ01-03, N02AJ05-09, N02AC06, N02AC52

Antineuropathic drugs

ATC codes N03AX12, N03AX16, N06AA09, N06AX21, N06AX16, N06AX11, N06AA12, N03AF01, N03AF02, N03AX09, N02BG10

Cortisone

ATC codes H02AB06, H02AB07

Bisphosphonates

ATC codes M05BA01-08

[1]OPS: Operationen- und Prozeduren Schlüssel (German Procedure Codes) is the official classification for coding operations, procedures, and general medical measures in the German health care system.
[2]EBM: Einheitlicher Bewertungsmaßstab (Uniform Value Scale) defines the content of billable services provided by accredited physicians/therapists in the German health care system.

Supplement 2. Percentage of CRPS cases in relation to the total number of patients per age decade.

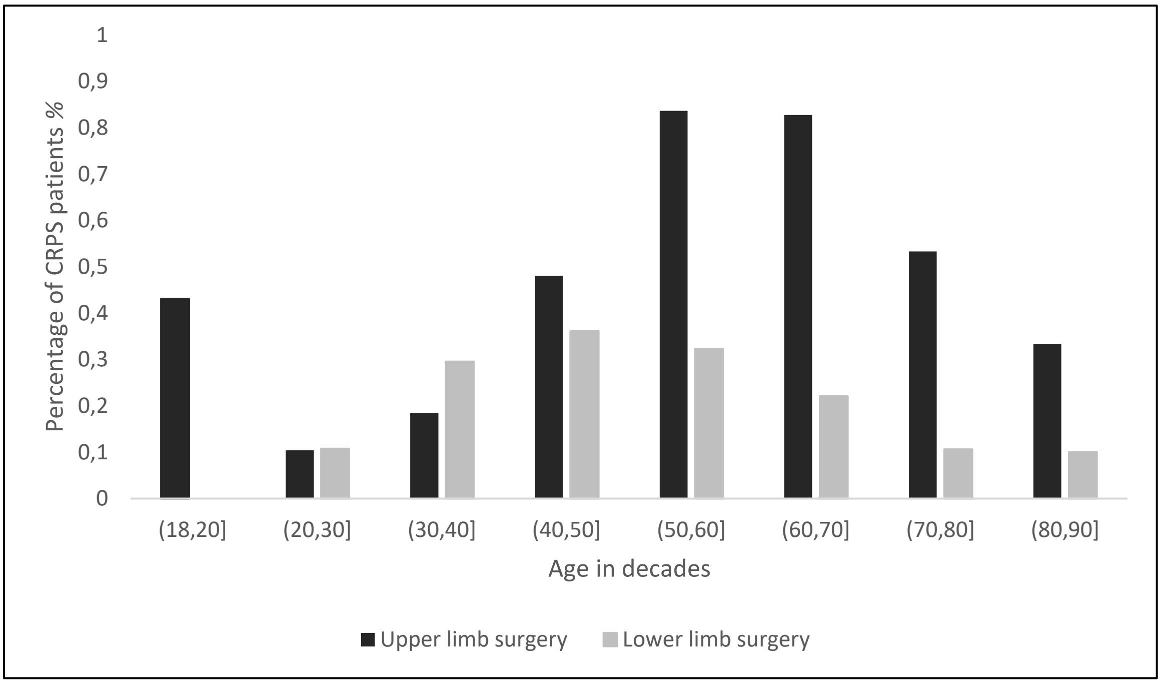

Supplement: Supplementary file 1 [file painreports-9-e1210-s001.pdf]
